# Phenotypic and Genotypic Characterization of Multidrug-Resistant Enterobacter hormaechei Carrying *qnrS* Gene Isolated from Chicken Feed in China

**DOI:** 10.1128/spectrum.02518-21

**Published:** 2022-04-25

**Authors:** Zhengzheng Cao, Luqing Cui, Quan Liu, Fangjia Liu, Yue Zhao, Kaixuan Guo, Tianyu Hu, Fan Zhang, Xijing Sheng, Xiangru Wang, Zhong Peng, Menghong Dai

**Affiliations:** a The Cooperative Innovation Center for Sustainable Pig Production, Huazhong Agricultural University, Wuhan, China; b MOA Key Laboratory of Food Safety Evaluation/National Reference Laboratory of Veterinary Drug Residue (HZAU), Huazhong Agricultural University, Wuhan, China; University of Adelaide

**Keywords:** *Enterobacter hormaechei*, *qnrS*, chicken feed, IncF, multidrug resistance, transfer, gene transfer

## Abstract

Multidrug resistance (MDR) in Enterobacteriaceae including resistance to quinolones is rising worldwide. The plasmid-mediated quinolone resistance (PMQR) gene *qnrS* is prevalent in Enterobacteriaceae. However, the *qnrS* gene is rarely found in Enterobacter hormaechei (*E. hormaechei*). Here, we reported one multidrug resistant *E. hormaechei* strain M1 carrying the *qnrS1* and *bla*_TEM-1_ genes. This study was to analyze the characteristics of MDR *E. hormaechei* strain M1. The *E. hormaechei* strain M1 was identified as Enterobacter cloacae complex by biochemical assay and 16S rRNA sequencing. The whole genome was sequenced by the Oxford Nanopore method. Taxonomy of the *E. hormaechei* was based on multilocus sequence typing (MLST). The *qnrS* with the other antibiotic resistance genes were coexisted on IncF plasmid (pM1). Besides, the virulence factors associated with pathogenicity were also located on pM1. The *qnrS1* gene was located between insertion element IS2A (upstream) and transposition element ISKra4 (downstream). The comparison result of IncF plasmids revealed that they had a common plasmid backbone. Susceptibility experiment revealed that the *E. hormaechei* M1 showed extensive resistance to the clinical antimicrobials. The conjugation transfer was performed by filter membrane incubation method. The competition and plasmid stability assays suggested the host bacteria carrying *qnrS* had an energy burden. As far as we know, this is the first report that *E. hormaechei* carrying *qnrS* was isolated from chicken feed. The chicken feed and poultry products could serve as a vehicle for these MDR bacteria, which could transfer between animals and humans through the food chain. We need to pay close attention to the epidemiology of *E. hormaechei* and prevent their further dissemination.

**IMPORTANCE** Enterobacter hormaechei is an opportunistic pathogen. It can cause infections in humans and animals. Plasmid-mediated quinolone resistance (PMQR) gene *qnrS* can be transferred intergenus, which is leading to increase the quinolone resistance levels in Enterobacteriaceae. Chicken feed could serve as a vehicle for the MDR *E. hormaechei*. Therefore, antibiotic-resistance genes (ARGs) might be transferred to the intestinal flora after entering the gastrointestinal tract with the feed. Furthermore, antibiotic-resistant bacteria (ARB) were also excreted into environment with feces, posing a huge threat to public health. This requires us to monitor the ARB and antibiotic-resistant plasmids in the feed. Here, we demonstrated the characteristics of one MDR *E. hormaechei* isolate from chicken feed. The plasmid carrying the *qnrS* gene is a conjugative plasmid with transferability. The presence of plasmid carrying antibiotic-resistance genes requires the maintenance of antibiotic pressure. In addition, the *E. hormaechei* M1 belonged to new sequence type (ST). These data show the MDR *E. hormaechei* M1 is a novel strain that requires our further research.

## INTRODUCTION

Enterobacter hormaechei is a member of the E. cloacae complex and has different subspecies (1). *E. hormaechei* is widespread in the environment, as a nosocomial pathogen causing neonatal bloodstream and urinary infections (2, 3). It also causes infections in animals. For example, *E. hormaechei* brought about respiratory disease for calves and septic arthritis in a green sea turtle (4, 5). Moreover, *E. hormaechei* strains that showed resistance to the major clinical antibiotics were isolated from the cloacae of poultry and a hand washing sink of a veterinary hospital (6, 7). *QnrS*, plasmid-mediated quinolone resistance (PMQR) gene, encodes for a protein of the pentapeptide repeat family that protects DNA gyrase and topoisomerase IV from quinolones inhibition (8).

PMQR genes are prevalent in Enterobacteriaceae, especially Escherichia coli, Klebsiella, Salmonella, and some species of Enterobacter (9–17). *QnrS* usually coexisted with multiple antibiotic resistance genes in the same plasmid to mediate multidrug resistance (MDR) (18–20). Currently, *E. hormaechei* carrying the *qnrS* gene has been reported (21). Moreover, our recent study showed that E. coli carrying the *qnrS* gene were isolated from chicken feed (22). However, the contamination of *E. hormaechei* in the feed has not been reported.

In this study, we aimed to analyze characteristics of *E. hormaechei* strain M1 carrying the *qnrS1* gene from the chicken feed, including the resistance level of *E. hormaechei* strain M1, transferability of plasmid, molecular traits of plasmid, as well as the genetic context of the *qnrS1* gene.

## RESULTS

### The identification of *E. hormaechei* M1 isolated from chicken feed.

The conventional biochemical tests were used to identify *E. hormaechei* M1. Through sugar and alcohol metabolism, amino acid and protein metabolism, carbon and nitrogen source utilization, and enzyme tests (Table 1), it was preliminarily determined to be *E. hormaechei*.

**TABLE 1:** Biochemical identification of *E. hormaechei* M1

Test	Result^*a*^	Test	Result
Maltose	+	Citrate	+
Glucose	+	Indole	−
Lactose	−	Voges-Proskauer	−
Sucrose	+	Methyl red	−
Arabinose	+	Ornithine decarboxylase	+
Mannose	+	Malonate	+
Mannitol	+	D- Sorbitol	+
Dulcitol	−	D- Arabitol	−
α-D- Melibiose	+	Adonitol	−

a +, positive; −, negative.

### Analysis of genome characteristics of *E. hormaechei* M1.

Sequencing results generated a complete chromosome of 4,658,400 bp and a plasmid 148,434 bp. The *oqxA*, *oqxB*, *bacA*, *bla*_ACT-25_, and *fosA2* genes were located on chromosome. The *catA2*, *tet(D)*, *dfrA14*, *aph(3′)-Ib*, *aph(*6*)-Id*, *qnrS1*, *sul2*, *bla*_TEM-1_ genes coexisted in the same plasmid; the plasmid incompatibility group was IncF, it had two replicons IncFII and IncFIB.

### Characterization of plasmids.

**FIG 1:**
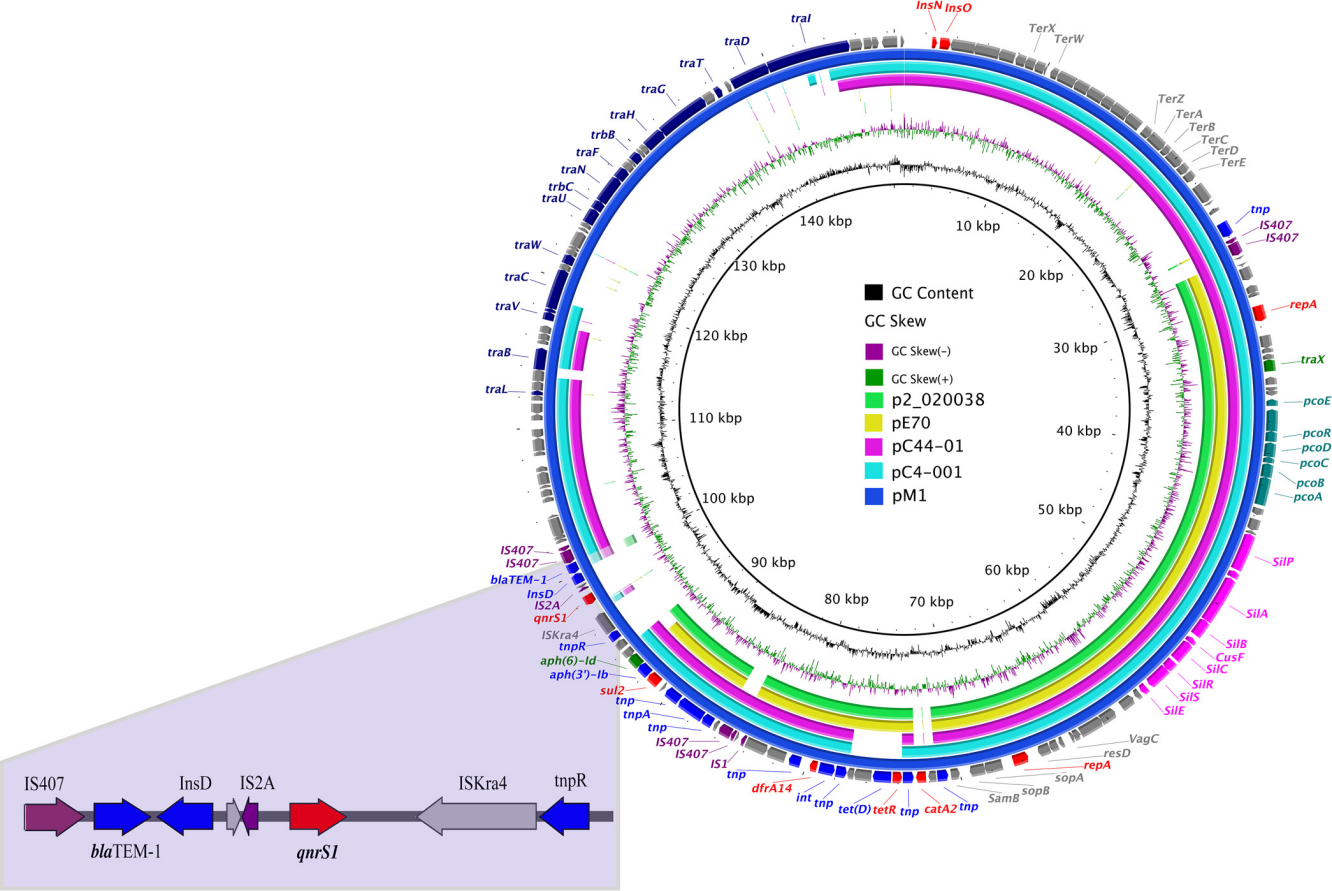
The complete sequence of pM1 (the outer circle) was used as a reference plasmid. The circular maps were generated using the BRIG software, and plasmids were included in the following order (inner to outer circles): p2-020038 (GenBank ID: CP031723.1), pE70 (CP046273.1), pC44-01 (CP042567.1), pC4-001 (CP042541.1), and pM1 (CP090910).

The circular image and circular comparisons between pM1 and other reported similar IncF plasmids were completed using the BRIG tool (Fig. 1). Plasmids were included in the following order (inner to outer circles): p2-020038, pE70, pC44-01, pC4-001, and pM1. These plasmids harbored the IncFII and IncFIB replicons. However, the pM1 had unique genes, such as antibiotic resistance genes (*qnrS1* and *bla*_TEM-1_), insertion sequences (InsD and IS2A), transposable elements (ISKra4 and tnpR), and pili operon (*traD*, *traF*, *traN*, *traU*, *traW*, *traT*, *traG*, *traH*, *trbB* and *trbC*). Meanwhile, these plasmids all harbored the *sul2* (sulfonamides), *dfrA14* (trimethoprim), *aph(3”)-Ib* (aminoglycosides), *aph(*6*)-Id* (aminoglycosides) resistance genes, and copper and silver heavy metal resistance genes. Moreover, the features were related to virulence, for example, copper and silver metal cation and tellurium ion efflux system, sopA and sopB, virulence-associated protein VagC, fimbrial operon, type IV pili and the hok toxic. The pM1 had a size of 148,434 bp with an average G+C content of 52%, and carried the *qnrS*1 gene that was located within the physical boundaries demarcated by insertion element IS2A (upstream) and transposition element ISKra4 (downstream). A genetic structure surrounding the *qnrS1* gene was detected from pM1(IS407-*bla*_TEM-1_-IS2A-*qnrS1*-ISKra4-tnpR).

### Analysis of phylogenetic tree.

**FIG 2:**
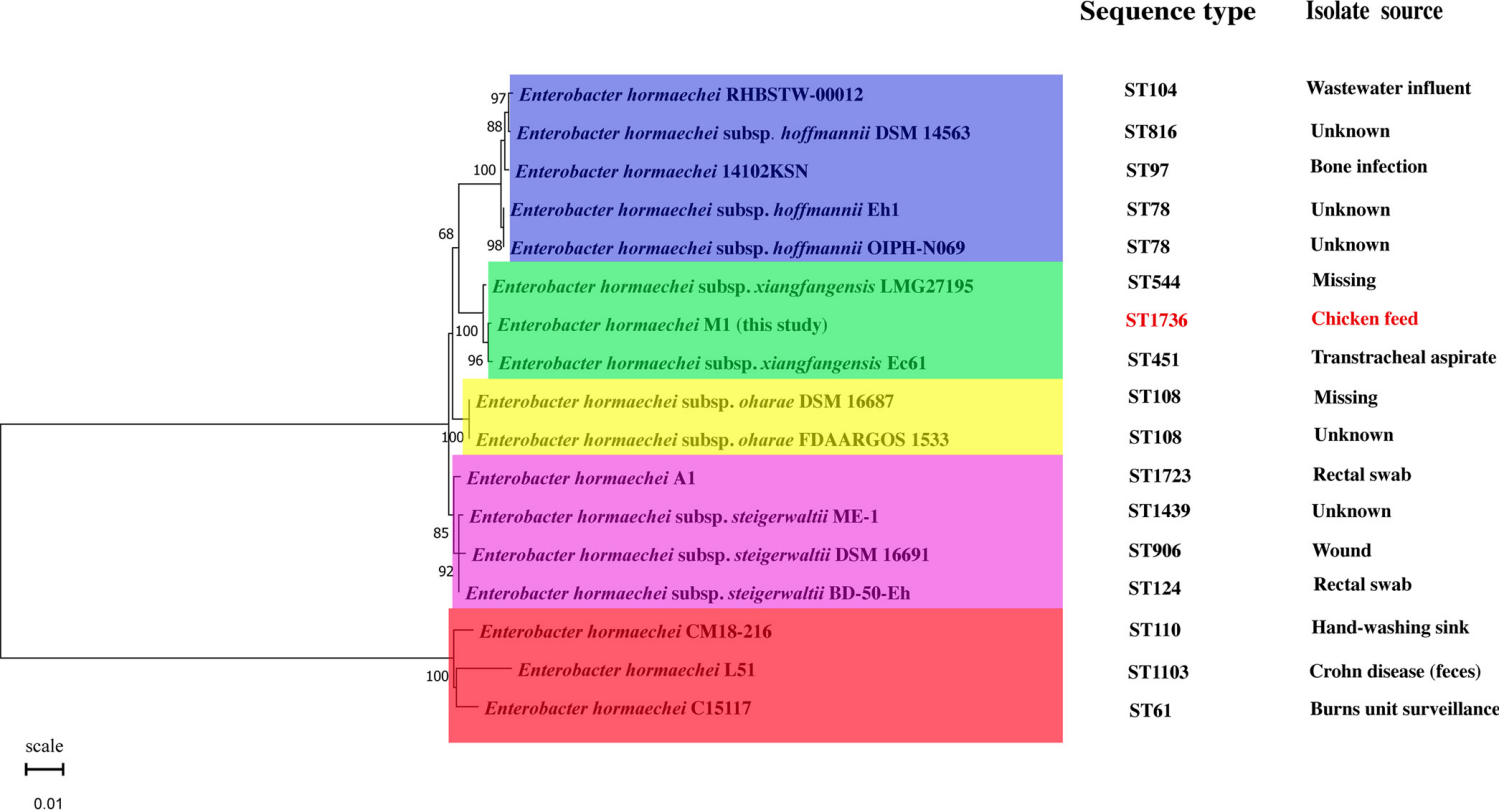
The evolutionary history was inferred by using the maximum likelihood method and Hasegawa-Kishino-Yano model. The tree with the highest log likelihood (–7,997.87) is shown. The percentage of trees in which the associated taxa clustered together is shown next to the branches. Initial tree(s) for the heuristic search were obtained automatically by applying Neighbor-Join and BioNJ algorithms to a matrix of pairwise distances estimated using the maximum composite likelihood (MCL) approach, and then selecting the topology with superior log likelihood value. A discrete Gamma distribution was used to model evolutionary rate differences among sites (five categories [+G, parameter = 0.2152]). The tree is drawn to scale, with branch lengths measured in the number of substitutions per site. This analysis involved 17 nucleotide sequences. All positions containing gaps and missing data were eliminated (complete deletion option). There was a total of 3,473 positions in the final data set. Evolutionary analyses were conducted in MEGA11.

The *dnaA*, *fusA*, *gyrB*, *leuS*, *pyrG*, *rplB*, and *rpoB* 7 housekeeping genes were determined by multilocus sequence typing (MLST) (https://pubmlst.org/). The seven genes were concatenated to make a phylogenetic tree (Fig. 2). The 17 strains of *E. hormaechei* were divided into five different groups and distinguished by different colors. Sequence type (ST) and isolate source of the strain were listed in tree. The phylogenetic tree showed that the *E. hormaechei* M1 was divided into Enterobacter hormaechei subsp. *xiangfangensis* group. It was closely related to the evolution of Enterobacter hormaechei isolated from human.

### Antimicrobials resistance of *E. hormaechei* M1 strain.

Susceptibility testing revealed that *E. hormaechei* M1 was resistant to most of the antimicrobials tested (Table 2). *E. hormaechei* M1 showed resistance to the following antimicrobials: β-lactams antibiotics (ampicillin, amoxicillin and cefalexin), quinolones (enrofloxacin), chloramphenicol, florfenicol, tetracyclines (oxytetracycline, tetracycline and doxycycline), sulfonamides (sulfadiazine), fosfomycin, compound medicines (amoxicillin/clavulanate potassium, trimethoprim-sulfamethoxazole). However, *E. hormaechei* M1 was sensitive to meropenem, gentamicin and polymyxin.

**TABLE 2:** MIC of 18 antimicrobials to *E. hormaechei* M1

Antimicrobials	MIC (μg/mL)	Results^*a*^
Ampicillin	>64	R
Amoxicillin	>64	R
Cefalexin	>64	R
Ceftriaxone	2	I
Meropenem	0.06	S
Enrofloxacin	4	R
Ciprofloxacin	2	I
Chloramphenicol	>128	R
Florfenicol	16	R
Oxytetracycline	>64	R
Tetracycline	>64	R
Doxycycline	64	R
Amoxicillin/clavulanate potassium	>32/16	R
Trimethoprim/sulfamethoxazole	>4/76	R
Sulfadiazine	>1024	R
Gentamicin	0.5	S
Polymyxin	0.06	S
Fosfomycin	256	R

a R, resistance; I, intermediate; S, sensitive.

### The conjugation transfer efficiency and the characteristics of transconjugants resistance.

The conjugation transfer experiment showed that donor *E. hormaechei* M1 strain transferred the plasmid pM1 to the recipient E. coli C600 strain. Conjugation transfer efficiency was $4.6\times 10^{-5}\pm 4.1\times 10^{-5}$. The susceptibility results of the recipient strain and the transconjugant showed that the transconjugant not only acquired *catA2*, *tet(D)*, *dfrA14*, *aph(3′)-Ib*, *aph(*6*)-Id*, *qnrS1*, *sul2*, and *bla*_TEM-1_ resistance genes but also emerged the corresponding resistance phenotype. The MIC of the ampicillin and tetracycline, enrofloxacin, ciprofloxacin, and ceftriaxone to the transconjugant were 16, eight, and two times, respectively, that of the recipient strain but the MIC of gentamicin was the same. The transconjugant was also sensitive to gentamicin (Table 3).

**TABLE 3:** Antimicrobials susceptibility determination of transconjugant and recipient stains

Strains	Minimum inhibitory concn of antimicrobials (μg/mL)								
	AMP^*a*^	SMZ	ACP	GEN	CHL	TET	ENR	CIP	CTRX
E. coli C600	4	0.25/4.75	16/8	0.5	2	4	0.25	1	0.25
E. coli C600-pM1	64	>4/76	>64/32	0.5	>128	64	2	2	0.5

a AMP, ampicillin; SMZ, trimethoprim-sulfamethoxazole; ACP, amoxicillin/clavulanate potassium; GEN, gentamicin; CHL, chloramphenicol; TET, tetracycline; ENR, enrofloxacin; CIP, ciprofloxacin; CTRX, ceftriaxone.

### Fitness cost and plasmid stability.

**FIG 3:**
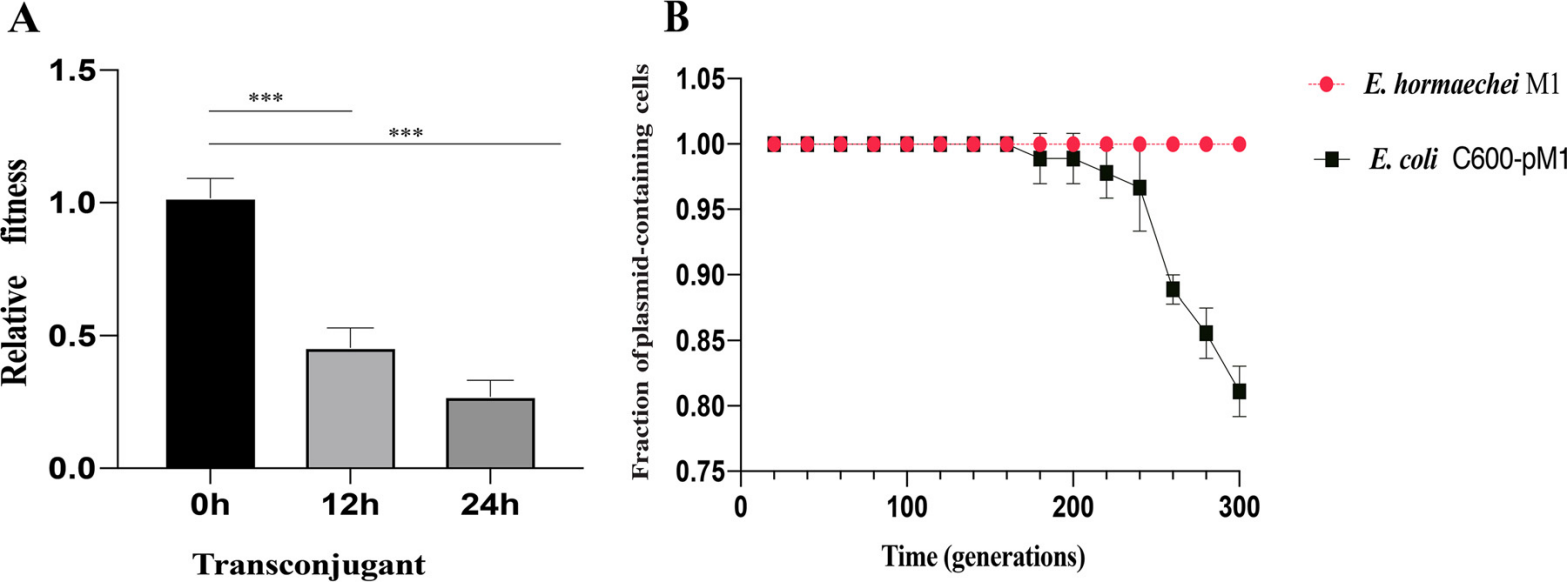
The (A) figure shows relative fitness of transconjugant carrying *qnrS1*. A relative fitness of 1 indicates that the conjugant underwent no fitness cost. The conjugant showed a relative fitness of 0.27 ± 0.047 (0 h versus 24 h, *P* < 0.01) in this study. The (B) figure shows plasmid lost in serial passages without antibiotic selection pressure.

The transconjugant carrying *qnrS1*-positive plasmid originated from *E. hormaechei* M1 showed a relative fitness of 0.27 ± 0.047 (0 h VS. 24 h, *P* < 0.01). This result showed that the acquisition of *qnrS1*-bearing plasmid could place an energy burden on the bacterial host and incur fitness cost (Fig. 3A). Plasmid stability result showed that the transconjugant (E. coli C600-pM1) could be steadily passaged to 160 generations without antibiotic stress, then the plasmid was gradually lost. The plasmid containing rates in the 180 and 200; 220; 240; 260; 280; and 300 generations were 0.99 ± 0.019; 0.98 ± 0.019; 0.97 ± 0.033; 0.89 ± 0.011; 0.86 ± 0.019; and 0.81 ± 0.019, respectively. The plasmid carried by *E. hormaechei* M1 was not lost (Fig. 3B).

## DISCUSSION

The MDR *E. hormaechei* was reported in humans (28–31), animals (4–6, 32), and the environment (33, 34). The prevalence of MDR Enterobacter spp. carrying genes of extended-spectrum β-lactamases (ESBLs) and PMQR has been increasing worldwide (35). The isolation of MDR *E. hormaechei* from the cloaca of poultry has been reported (6). Furthermore, *E. hormaechei* strains have been isolated from animal feces (36). They suggested that fecal excretion is one of the reasons for the prevalence of MDR bacteria. The PMQR gene *qnrS* is prevalent in chicken feces (22, 37, 38). The *qnrS* gene was also found in the chicken feed (22). In this study, *E. hormaechei* carrying the *qnrS* gene was isolated from chicken feed. We speculate that it may result from the contamination caused by the exposure of the feed in the chicken farm to the environment. Previous study has shown that antibiotic-resistant bacteria carried in feed can transfer resistance genes to the gut microbiota after entering the zebrafish gut (39). Therefore, *E. hormaechei* carried in chicken feed may also transfer resistance genes to intestinal flora.

The isolation of MDR *E. hormaechei* from chicken feed has not been previously reported. This study firstly reported that *E. hormaechei* carrying *qnrS1* and *bla*_TEM-1_ genes was existed in chicken feed. The results of biochemical experiments were consistent with the description of *E. hormaechei*. The phenotypic-based tests were not accurate enough for species identification. Therefore, molecular identification was required (40). The MLST is a powerful tool for resolving the phylogeny of closely related species of the genus. The MLST especially suits to resolving complex phenotypes, such as virulence and antibiotic resistance in bacterial pathogens (41). The current reports about sequence types of *E. hormaechei* are ST78, ST873, ST66, ST419, ST145, ST50, ST118, and ST168 (42–44). However, the sequence type of *E. hormaechei* M1 was ST1736, which belonged to a new ST.

The *E. hormaechei* M1 carried IncF plasmid. Usually, IncF plasmids can encode several replicons, which is a kind of typical multireplicon IncF plasmid carrying the FII replicon together with FIA and FIB (45). IncF plasmid relaxase type is MOB_F_, with the size from 45 to 200 kb, low copy number, conjugative, and most exist in Enterobacteriaceae (46). The functions of the proteins encoding the 40-kb *tra* operon were involved in formation of the F-type pilus (47). The conjugation transfer experiments and sequencing results also confirmed this point. The previous report showed that the *qnrS1* gene upstream element was IS2 (48). In this study, the *qnrS1* gene upstream element was IS2A which is a variant of IS2. More examples of a single IS mobilizing an adjacent region that includes one or more resistance genes are being identified, particularly in Gram-negative bacteria (49). In this investigation, we also found the IS407 and IS2A insertion elements were located upstream of *bla*_TEM-1_ and *qnrS1* genes. Furthermore, the insertion elements upstream of antibiotic resistance genes (ARGs) affected antibiotic resistance phenotypes (50). The *E. hormaechei* M1 was low-level resistance to ciprofloxacin, which might be related to the upstream insertion element of *qnrS* gene. *QnrS* usually with *bla*_TEM_ coexisted on the same plasmid (18, 51–53). In this research, the *qnrS1*, *bla*_TEM-1_, *tet(D)*, *sul2*, *catA2*, *dfrA14*, *aph(3”)-Ib*, and *aph(*6*)-Id* coexisted on pM1. Although pM1 harbored *aph(3”)-Ib* and *aph(*6*)-Id* aminoglycosides resistance genes, it did show sensitive to gentamicin, which might be due to the mutations affecting the *aph(3”)-Ib* and *aph(*6*)-Id* genes or their promoter region (54).

The plasmid stability experiment suggests that plasmid will be lost in the absence of antibiotic selection pressure. Similarly, the plasmid carrying the *bla*_NDM-5_ gene was lost in E. coli by serial passage on medium without antibiotics (55), which may be attributed to non-selective conditions, for example, temperature and transcription, and replication and DNA topology that lead to plasmid instability (56). In addition, the competition between transcription and replication of the same DNA template leads to increased mutations and reduces the integrity of the genome due to replication and deletion events (57), which incurs plasmid loss. The previous report showed that the proportion of strains carrying plasmids with antibiotic resistance genes were decreased in absence selective pressure (58). Our research also found that the proportion of transconjugant (E. coli C600-pM1) was decreased in competition experiments. When the cost of plasmid carriage outweighs its benefit, plasmid-free proportion are expected to outcompete plasmid-carrying bacteria, eventually leading to plasmid loss (59). Plasmid maintenance is considered a metabolic burden to the host bacteria (60). Similarly, the transconjugant carrying pM1 showed a decreased fitness. Antibiotics may be a factor in maintaining the long-term stable existence of resistant plasmids.

The *E. hormaechei* strain carrying pC44-01 and pC4-001 was isolated from clinical samples of an Australian hospital (61). The *E. hormaechei* strain WCHEH020038 carrying p2_020038 was isolated from Center of Infectious Diseases, West China Hospital, Sichuan University. The *E. hormaechei* strain E70 carrying pE70 was isolated from a patient’s urine sample. Previous reports show that the isolation of *E. hormaechei* are mainly focused on clinical samples (2, 62, 63). In this study, the *E. hormaechei* strain M1 strain was isolated from chicken feed, which is a new source. The comparison of IncF plasmids showed that pC44-01 and pC4-001 had higher similarity with pM1 but lacked encoding fimbriae genes. The p2_020038, pE70, pC44-01, pC4-001, and pM1 common regions encoded samB (DNA damage repair), sopA, and sopB (plasmid partitioning related genes); resD (resolvase); copper and silver cation efflux; IncFII and IncFIB plasmid replicons; and *traX* (transfer) genes, which could define the general IncF backbone. It was worth studying the functions of virulence factors located on pM1, such as, cation efflux system; sopA and sopB; VagC; and fimbriae and hok toxic protein. The cation efflux system is related to virulence and might contribute to bacteria in environmental persistence and host colonization. Furthermore, bacterial influx systems for essential trace cations are known to contribute to pathogenesis (64–66). The VagC protein has been indirectly implicated in plasmid maintenance. The VagCD proteins were proved as functional TA systems with VagD the toxin and VagC its antitoxin (67, 68). The pM1 also carried the *VagCD* genes that may contribute to pathogenicity of *E. hormaechei*. Fimbriae act as independent virulence factors by promoting the establishment of bacteria and the innate host response, which is responsible for symptoms and tissue damage (69). The pM1 had more fimbriae encoding genes than other plasmids. It is possible that *E. hormaechei* M1 is highly pathogenic to the host. The *hok/sok* locus is a type I toxin/antitoxin plasmid stability element that increases bacterial tolerance to β-lactam antibiotics, which enhances bacteria survivability and pathogenicity in stressful growth conditions (70). These virulence factors allow bacteria to survive better under stress conditions. We also need to further explore the functions of these virulence factors to better reveal the pathogenic mechanism of *E. hormaechei*. In conclusion, MDR *E. hormaechei* in feed might be a risk control point of transmission of resistance genes. It is necessary to continuously strengthen the monitoring of MDR bacteria in feed and take effective measures to eliminate resistance genes and plasmids, so as to prevent the spread of resistance genes from aggravating clinical drug resistance.

### Conclusion.

This is the first report that *E. hormaechei* carrying *qnrS* and *bla*_TEM-1_ has been isolated from chicken feed. It is a MDR *E. hormaechei* strain. Importantly, the plasmid pM1 not only contained multiple resistance genes but also virulence factors related to its pathogenicity. The concern is that chicken feed and poultry products could serve as a vehicle for these MDR bacteria, which could be transferred between animals and humans through the food chain. The IncF of conjugation plasmids could transfer intergenus. If pathogenic bacteria acquire the drug-resistant plasmids, it will lead to the failure of clinical treatment. The MDR *E. hormaechei* may be a public health issue. There is an urgent need for close epidemiologic surveillance to control their further spread.

## MATERIALS AND METHODS

### Isolation and identification of strains.

The *E. hormaechei* strain M1 was isolated from feed of a chicken farm in Hubei Province, China. The feed samples were added to 4 mL LB broth (Hopebio, Qingdao, China) and incubated at 37°C for 4 h to 5 h, then 100-μL cultures were spread on the LB agar plates supplemented with enrofloxacin (1 μg/mL) overnight at 37°C. The single colonies were randomly selected from the LB agar plates and identified by 16S rRNA sequencing. Meanwhile, the *E. hormaechei* strain M1 was identified by conventional biochemical tests.

### Reagents.

The standards of enrofloxacin, amoxicillin, tetracycline, chloramphenicol, ampicillin, trimethoprim, florfenicol, doxycycline, gentamicin, oxytetracycline, sulfadiazine, and sulfamethoxazole were purchased from Dr. Ehrenstorfer (Germany). Clavulanate potassium, meropenem, polymyxin, fosfomycin, cefalexin, and ceftriaxone were obtained from China National Institutes for Drug Control. Rifampicin was purchased from Shanghai Yuanye Bio-Technology Co., Ltd. Ciprofloxacin standards was purchased by MedChemExpress (New Jersey, USA). The 2 × EasyTaq PCR Master Mix (Dye plus) and Phanta super-fidelity DNA polymerase were purchased from Vazyme (Nanjing, China). Bacterial Micro Biochemical Tubes were bought from Hangzhou Microbial Regent Co., Ltd and Hopebio, Qingdao, China.

### Whole genome sequencing and molecular analysis of E. *hormaechei* M1.

*E. hormaechei* strain M1 was collected by propagating culture in LB broth (Hopebio, Qingdao, China), centrifuged and frozen in liquid nitrogen. Whole genome sequencing (WGS) was done by Oxford Nanopore Technologies DNA sequencing platform (Biomarker Technologies, Beijing, China). The genes were automatically annotated by Prodigal v2.6.3. Then manual inspection and correction used the BLASTn and BLASTp programs (https://blast.ncbi.nlm.nih.gov/Blast.cgi). Plasmid type comparison of PlasmidFinder was carried out on (http://www.genomicepidemiology.org/) and the resistance genes encoded proteins were blasted by Uniprot (https://www.uniprot.org/). Alignments of similar IncF plasmids were created by BRIG tools (https://sourceforge.net/projects/brig/). The accession numbers of the plasmids were as follows: p2-020038 (CP031723.1), pE70(CP046273.1), pC44-01 (CP042567.1), pC4-001 (CP042541.1), and pM1 (CP090910).

### Phylogenetic tree.

The *E. hormaechei* M1 (accession number CP090909) genome was uploaded onto MLST (https://pubmlst.org/). Then the *dnaA*, *fusA*, *gyrB*, *leuS*, *pyrG*, *rplB*, and *rpoB* housekeeping genes were determined (23). Taxonomic evaluation of the *E. hormaechei* based on MLST. The phylogenetic tree of 17 strains of *E. hormaechei* was produced by MEGA11 (https://www.megasoftware.net/). The accession numbers of these strains were as follows: DSM 16691 (CP017179.1), DSM16687 (CP017180.1), L51 (CP033102.1), RHBSTW-00012 (CP058191.1), A1 (CP031574.1), 14102KSN (CP045312.1), LMG27195 (CP017183.1), Ec61 (CP053103.1), DSM14563 (CP017186.1), Eh1 (CP034754.1), OIPH-N069 (AP019817.1), FDAARGOS_1533 (CP083613.1), ME-1 (CP041733.1), and BD-50-Eh (CP063224.1) strains were isolated from humans. Moreover, the C15117 (CP032841.1) and CM18-216 (CP050311.1) stains were isolated from hospital environment. Their genomes were obtained by NCBI (https://www.ncbi.nlm.nih.gov/).

### Susceptibility tests.

The MICs of ampicillin, amoxicillin, cefalexin, enrofloxacin, ciprofloxacin, chloramphenicol, florfenicol, tetracyclines, oxytetracycline, tetracycline, doxycycline, sulfadiazine, fosfomycin, camoxicillin/clavulanate potassium, trimethoprim-sulfamethoxazole, meropenem, gentamicin, and polymyxin were measured by broth microdilution and agar dilution methods according to the recommendations of the CLSI document M100-30th ed (CLSI 2020) (24). According to the criteria of Enterobacteriaceae, the E. coli ATCC25922 strain was used as quality control strain. The susceptibility tests were repeated three times.

### Conjugation transfer experiment.

MacConkey plates were supplemented with 125 μg/mL rifampicin for counting recipient bacteria, and with 125 μg/mL rifampicin and 2 μg/mL enrofloxacin for counting the conjugants. *E. hormaechei* M1 and E. coli C600 were used as donor and recipient strain, respectively. They were subcultured on eosin methylene blue (EMB) plates, and a single colony was selected and inoculated into 1 mL LB broth and cultured with shaking at 37°C for 5 h to 6 h. The 0.22-μm filter membrane was placed on the antibiotic-free LB agar medium. Then, 20 μL of the donor bacteria and 60 μL of the recipient bacteria were mixed in an EP tube, which were equably aliquoted onto the filter membrane and incubated overnight at 37°C. The bacterial lawn was washed down with 1 mL of antibiotic-free LB broth, the 100-μL bacterial solution was proceeded with multiple dilution. Bacterial solution with a dilution of 101:1,010 was spread on the LB agar plates supplemented with 125 μg/mL rifampicin and 2 μg/mL enrofloxacin, then the plates were selected from the colonies between 30 and 300 for counting. The bacterial solution with a dilution of 101:1,010 was spread on the LB agar supplemented with 125 μg/mL rifampicin plates respectively, and the plates were selected from the colonies between 30 and 300 for counting. The conjugation transfer efficiency = number of transconjugants/number of recipients Then, five conjugants were randomly selected from the LB agar plates supplemented with 125 μg/mL rifampicin and 2 μg/mL enrofloxacin, and their homology with the recipient strain E. coli C600 were verified by ERIC-PCR.

### Plasmid stability tests.

To estimate plasmid stability, E. coli C600 conjugants and *E. hormaechei* M1 carrying *qnrS* were cultured in antibiotic-free LB broth, respectively, overnight at 37°C. The cultures were diluted 1:10^3^ in fresh LB medium and were further incubated overnight at 37°C. Serial passaging of 1 μL of overnight culture to 1 mL of LB was performed daily, approximately 10 generations per passage. Every 20 generations, the cultures were diluted and spread on LB agar plates. The ratio of colonies grown on antibiotic-supplemented LB agar (2 μg/mL enrofloxacin) compared with that on antibiotic-free LB agar was determined in triplicate. Presence of *qnrS* gene in the host after each passage was verified by PCR. The colonies grown on antibiotic-free/supplemented agar were randomly selected (~30 colonies per agar) as DNA template (25).

### Competition experiments to assess *in vitro* fitness.

To assess the fitness impact of *qnrS* gene carriage, pairwise competition assays were carried out using the E. coli C600 transconjugants carrying *qnrS* gene competed with its plasmid-free counterparts. As described previously, 24 h competition experiments were performed (26). Briefly, cultures were adjusted to a 1.0 McFarland standard, which were diluted 1:10^4^ and then mixed at a volumetric ratio of 1:1 (time point zero). Colony counts were determined by plating serial dilutions of mixed cultures on LB agar (LBA) with and without enrofloxacin (2 μg/mL) at 0 h, 12 h, and 24 h. The number of CFU growing on antibiotic-supplemented LBA was subtracted from the number of CFU growing on antibiotic-free LBA to determine the number of susceptible cells in the mixed population. All experiments were performed in triplicate and at least four replicates of each competition assay were performed. The relative fitness is calculated by the ratio of the growth rate of the resistant cells to that of the susceptible ones according to previous report (27). A relative fitness of 1 indicated that the transconjugants undergo no fitness cost, whereas a ratio of greater than or less than 1 indicated increased or decreased fitness, respectively.

### Statistical analysis.

Using GraphPad Prism 8.0 statistical software, the value was expressed by mean ± SD, and the difference between different time points was analyzed by one-way ANOVA. ***, *P* ≤ 0.01 (extremely significant difference).

### Data availability.

The whole genome sequences of *E. hormaechei* strain M1 were uploaded into NCBI databases. Their accession numbers are as follows: *E. hormaechei* M1 (CP090909), pM1 (CP090910).

## Supplementary Material

Reviewer comments

## References

[1] Hoffmann H, Roggenkamp A. 2003. Population genetics of the nomenspecies *Enterobacter cloacae*. Appl Environ Microbiol 69:5306–5318. doi:10.1128/AEM.69.9.5306-5318.2003. 12957918 PMC194928

[2] Martins ER, Bueno MFC, Francisco GR, Casella T, de Oliveira Garcia D, Cerdeira LT, Gerber AL, de Almeida LGP, Lincopan N, de Vasconcelos ATR, Nogueira MCL, Estofolete CF. 2020. Genome and plasmid context of two *rmtG*-carrying *Enterobacter hormaechei* isolated from urinary tract infections in Brazil. J Glob Antimicrob Resist 20:36–40. doi:10.1016/j.jgar.2019.06.020. 31279132

[3] da Silva CLP, Miranda LEV, Moreira BM, Rebello D, Carson LA, Kellum ME, de Almeida MCL, Sampaio JLM, O'Hara CM. 2002. *Enterobacter hormaechei* bloodstream infection at three neonatal intensive care units in Brazil. Pediatr Infect Dis J 21:175–177. doi:10.1097/00006454-200202000-00022. 11840092

[4] Wang Z, Duan L, Liu F, Hu Y, Leng C, Kan Y, Yao L, Shi H. 2020. First report of *Enterobacter hormaechei* with respiratory disease in calves. BMC Vet Res 16:1. doi:10.1186/s12917-019-2207-z. 31900161 PMC6942294

[5] Goldberg DW, Fernandes MR, Sellera FP, Costa DGC, Loureiro Bracarense AP, Lincopan N. 2019. Genetic background of CTX-M-15-producing *Enterobacter hormaechei* ST114 and Citrobacter freundii ST265 co-infecting a free-living green turtle (Chelonia mydas). Zoonoses Public Health 66:540–545. doi:10.1111/zph.12572. 30843359

[6] Nandi SP, Sultana M, Hossain MA. 2013. Prevalence and characterization of multidrug-resistant zoonotic *Enterobacter* spp. in poultry of Bangladesh. Foodborne Pathog Dis 10:420–427. doi:10.1089/fpd.2012.1388. 23560422

[7] Kamathewatta K, Bushell R, Rafa F, Browning G, Billman-Jacobe H, Marenda M. 2020. Colonization of a hand washing sink in a veterinary hospital by an *Enterobacter hormaechei* strain carrying multiple resistances to high importance antimicrobials. Antimicrobial Resistance and Infection Control 9:163. doi:10.1186/s13756-020-00828-0. 33087168 PMC7580002

[8] Jacoby GA, Strahilevitz J, Hooper DC. 2014. Plasmid-mediated quinolone resistance. Microbiol Spectr 2. doi:10.1128/microbiolspec.PLAS-0006-2013. PMC4288778 25584197

[9] Mahmud S, Nazir K, Rahman MT. 2018. Prevalence and molecular detection of fluoroquinolone-resistant genes (*qnrA* and *qnrS*) in isolated from healthy broiler chickens. Vet World 11:1720–1724. doi:10.14202/vetworld.2018.1720-1724. 30774264 PMC6362325

[10] Benaicha H, Barrijal S, Ezzakkioui F, Elmalki F. 2017. Prevalence of PMQR genes in *E. coli* and *Klebsiella* spp. isolated from North-West of Morocco. J Glob Antimicrob Resist 10:321–325. doi:10.1016/j.jgar.2017.05.024. 28735058

[11] Gosling RJ, Clouting CS, Randall LP, Horton RA, Davies RH. 2012. Ciprofloxacin resistance in *E. coli* isolated from turkeys in Great Britain. Avian Pathol 41:83–89. doi:10.1080/03079457.2011.640659. 22845325

[12] Nazir A, Zhao Y, Li M, Manzoor R, Tahir RA, Zhang X, Qing H, Tong Y. 2020. Structural genomics of *repA*, *repB*1-carrying IncFIB family pA1705- *qnrS*, P911021- *tetA*, and P1642- *tetA*, multidrug-resistant plasmids from *Klebsiella pneumoniae*. Infect Drug Resist 13:1889–1903. doi:10.2147/IDR.S228704. 32606838 PMC7319535

[13] Cattoir V, Weill F-X, Poirel L, Fabre L, Soussy C-J, Nordmann P. 2007. Prevalence of *qnr* genes in *Salmonella* in France. J Antimicrob Chemother 59:751–754. doi:10.1093/jac/dkl547. 17307773

[14] Cavaco LM, Aarestrup FM. 2009. Evaluation of quinolones for use in detection of determinants of acquired quinolone resistance, including the new transmissible resistance mechanisms *qnrA*, *qnrB, qnrS*, and *aac(6')Ib-cr*, in *Escherichia coli* and *Salmonella enterica* and determinations of wild-type distributions. J Clin Microbiol 47:2751–2758. doi:10.1128/JCM.00456-09. 19571019 PMC2738116

[15] Poirel L, Nguyen TV, Weintraub A, Leviandier C, Nordmann P. 2006. Plasmid-mediated quinolone resistance determinant *qnrS* in *Enterobacter cloacae*. Clin Microbiol Infect 12:1021–1023. doi:10.1111/j.1469-0691.2006.01531.x. 16961640

[16] Park Y-J, Yu JK, Lee S, Oh E-J, Woo G-J. 2007. Prevalence and diversity of *qnr* alleles in AmpC-producing *Enterobacter cloacae*, *Enterobacter aerogenes*, *Citrobacter freundii* and *Serratia marcescens*: a multicentre study from Korea. J Antimicrob Chemother 60:868–871. doi:10.1093/jac/dkm266. 17660263

[17] Wu J-J, Ko W-C, Tsai S-H, Yan J-J. 2007. Prevalence of plasmid-mediated quinolone resistance determinants QnrA, QnrB, and QnrS among clinical isolates of *Enterobacter cloacae* in a Taiwanese hospital. Antimicrob Agents Chemother 51:1223–1227. doi:10.1128/AAC.01195-06. 17242140 PMC1855486

[18] Li L, Wang B, Feng S, Li J, Wu C, Wang Y, Ruan X, Zeng M. 2014. Prevalence and characteristics of extended-spectrum β-lactamase and plasmid-mediated fluoroquinolone resistance genes in *Escherichia coli* isolated from chickens in Anhui province, China. PLoS One 9:e104356. doi:10.1371/journal.pone.0104356. 25141348 PMC4139264

[19] Chen Y-T, Shu H-Y, Li L-H, Liao T-L, Wu K-M, Shiau Y-R, Yan J-J, Su I-J, Tsai S-F, Lauderdale T-L. 2006. Complete nucleotide sequence of pK245, a 98-kilobase plasmid conferring quinolone resistance and extended-spectrum-beta-lactamase activity in a clinical *Klebsiella pneumoniae* isolate. Antimicrob Agents Chemother 50:3861–3866. doi:10.1128/AAC.00456-06. 16940067 PMC1635178

[20] Colobatiu L, Tabaran A, Flonta M, Oniga O, Mirel S, Mihaiu M. 2015. First description of plasmid-mediated quinolone resistance determinants and β-lactamase encoding genes in non-typhoidal *Salmonella* isolated from humans, one companion animal and food in Romania. Gut Pathog 7:16. doi:10.1186/s13099-015-0063-3. 26120367 PMC4482042

[21] Ai W, Zhou Y, Wang B, Zhan Q, Hu L, Xu Y, Guo Y, Wang L, Yu F, Li X. 2021. First report of coexistence of *bla*_SFO-1_ and *bla*_NDM-1_ *β*-lactamase genes as well as colistin resistance gene *mcr-9* in a transferrable plasmid of a clinical isolate of *Enterobacter hormaechei*. Front Microbiol 12:676113. doi:10.3389/fmicb.2021.741628. 34220761 PMC8252965

[22] Guo K, Zhao Y, Cui L, Cao Z, Zhang F, Wang X, Peng Z, Feng J, Hu T, Dai M. 2021. Longitudinal surveillance and risk assessment of resistance in *Escherichia coli* to enrofloxacin from a large-scale chicken farm in Hebei, China. Antibiotics (Basel, Switzerland) 10:1222. doi:10.3390/antibiotics10101222. 34680803 PMC8532996

[23] Jolley KA, Bray JE, Maiden MCJ. 2018. Open-access bacterial population genomics: BIGSdb software, the PubMLST.org website and their applications. Wellcome Open Res 3:124. doi:10.12688/wellcomeopenres.14826.1. 30345391 PMC6192448

[24] Clinical and Laboratory Standards Institute (CLSI). 2021. Performance standards for antimicrobial susceptibility testing, 30th ed. CLSI. https://clsi.org/.

[25] Sandegren L, Linkevicius M, Lytsy B, Melhus Å, Andersson DI. 2012. Transfer of an *Escherichia coli* ST131 multiresistance cassette has created a *Klebsiella pneumoniae*-specific plasmid associated with a major nosocomial outbreak. J Antimicrob Chemother 67:74–83. doi:10.1093/jac/dkr405. 21990049

[26] Lenski RE, Simpson SC, Nguyen TT. 1994. Genetic analysis of a plasmid-encoded, host genotype-specific enhancement of bacterial fitness. J Bacteriol 176:3140–3147. doi:10.1128/jb.176.11.3140-3147.1994. 8195066 PMC205481

[27] Gagneux S, Long CD, Small PM, Van T, Schoolnik GK, Bohannan BJM. 2006. The competitive cost of antibiotic resistance in *Mycobacterium tuberculosis*. Science 312:1944–1946. doi:10.1126/science.1124410. 16809538

[28] Mshana SE, Gerwing L, Minde M, Hain T, Domann E, Lyamuya E, Chakraborty T, Imirzalioglu C. 2011. Outbreak of a novel *Enterobacter* sp. carrying *bla*_CTX-M-15_ in a neonatal unit of a tertiary care hospital in Tanzania. Int J Antimicrob Agents 38:265–269. doi:10.1016/j.ijantimicag.2011.05.009. 21752606

[29] Ding M, Shi J, Ud Din A, Liu Y, Zhang F, Yan X, Li Q, Bai J, Chen W, Zhou Y. 2021. Co-infections of two carbapenemase-producing *Enterobacter hormaechei* clinical strains isolated from the same diabetes individual in China. J Medical Microbiology 70. doi:10.1099/jmm.0.001316. 33528353

[30] Mori N, Kagawa N, Aoki K, Ishi Y, Tateda K, Aoki Y. 2020. Clinical and molecular analyses of bloodstream infections caused by IMP metallo-β-lactamase-producing Enterobacteriaceae in a tertiary hospital in Japan. J Infect Chemother 26:144–147. doi:10.1016/j.jiac.2019.07.017. 31427199

[31] Rozales FP, Ribeiro VB, Magagnin CM, Pagano M, Lutz L, Falci DR, Machado A, Barth AL, Zavascki AP. 2014. Emergence of NDM-1-producing Enterobacteriaceae in Porto Alegre, Brazil. Int J Infect Dis 25:79–81. doi:10.1016/j.ijid.2014.01.005. 24857802

[32] Sidjabat HE, Hanson ND, Smith-Moland E, Bell JM, Gibson JS, Filippich LJ, Trott DJ. 2007. Identification of plasmid-mediated extended-spectrum and AmpC beta-lactamases in *Enterobacter* spp. isolated from dogs. J Med Microbiol 56:426–434. doi:10.1099/jmm.0.46888-0. 17314376

[33] Boutarfi Z, Rebiahi S-A, Morghad T, Perez Pulido R, Grande Burgos MJ, Mahdi F, Lucas R, Galvez A. 2019. Biocide tolerance and antibiotic resistance of *Enterobacter* spp. isolated from an Algerian hospital environment. J Glob Antimicrob Resist 18:291–297. doi:10.1016/j.jgar.2019.04.005. 31005732

[34] Fadare FT, Okoh AI. 2021. Distribution and molecular characterization of ESBL, pAmpC *β*-lactamases, and non-*β*-lactam encoding genes in Enterobacteriaceae isolated from hospital wastewater in Eastern Cape Province, South Africa. PLoS One 16:e0254753. doi:10.1371/journal.pone.0254753. 34288945 PMC8294522

[35] Kanamori H, Yano H, Hirakata Y, Hirotani A, Arai K, Endo S, Ichimura S, Ogawa M, Shimojima M, Aoyagi T, Hatta M, Yamada M, Gu Y, Tokuda K, Kunishima H, Kitagawa M, Kaku M. 2012. Molecular characteristics of extended-spectrum beta-lactamases and *qnr* determinants in *Enterobacter* species from Japan. PLoS One 7:e37967. doi:10.1371/journal.pone.0037967. 22719857 PMC3376121

[36] Ji Y, Wang P, Xu T, Zhou Y, Chen R, Zhu H, Zhou K. 2021. Development of a one-step multiplex PCR assay for differential detection of four species (*Enterobacter cloacae*, *Enterobacter hormaechei*, *Enterobacter roggenkampii*, and *Enterobacter kobei*) belonging to *Enterobacter cloacae* complex with clinical significance. Front Cell Infect Microbiol 11:677089. doi:10.3389/fcimb.2021.677089. 34095000 PMC8169972

[37] Murase T, Phuektes P, Ozaki H, Angkititrakul S. 2022. Prevalence of *qnrS*-positive *Escherichia coli* from chicken in Thailand and possible co-selection of isolates with plasmids carrying *qnrS* and trimethoprim-resistance genes under farm use of trimethoprim. Poult Sci 101:101538. doi:10.1016/j.psj.2021.101538. 34788713 PMC8591490

[38] Yang Y, Xie X, Tang M, Liu J, Tuo H, Gu J, Tang Y, Lei C, Wang H, Zhang A. 2020. Exploring the profile of antimicrobial resistance genes harboring by bacteriophage in chicken feces. Sci Total Environ 700:134446. doi:10.1016/j.scitotenv.2019.134446. 31648121

[39] Fu J, Yang D, Jin M, Liu W, Zhao X, Li C, Zhao T, Wang J, Gao Z, Shen Z, Qiu Z, Li J-W. 2017. Aquatic animals promote antibiotic resistance gene dissemination in water via conjugation: Role of different regions within the zebra fish intestinal tract, and impact on fish intestinal microbiota. Mol Ecol 26:5318–5333. doi:10.1111/mec.14255. 28742284

[40] Wu W, Feng Y, Zong Z. 2020. Precise species identification for *Enterobacter*: a genome sequence-based study with reporting of two novel species, *Enterobacter quasiroggenkampii* sp. nov. and *Enterobacter quasimori* sp. nov. mSystems 5. doi:10.1128/mSystems.00527-20. PMC7406230 32753511

[41] Vasemägi A, Primmer CR. 2005. Challenges for identifying functionally important genetic variation: the promise of combining complementary research strategies. Mol Ecol 14:3623–3642. doi:10.1111/j.1365-294X.2005.02690.x. 16202085

[42] Villa J, Carretero O, Viedma E, Lora-Tamayo J, Mingorance J, Chaves F. 2019. Emergence of NDM-7-producing multi-drug-resistant *Enterobacter hormaechei* sequence type ST-78 in Spain: a high-risk international clone. Int J Antimicrob Agents 53:533–534. doi:10.1016/j.ijantimicag.2018.11.009. 30472289

[43] Emeraud C, Petit C, Gauthier L, Bonnin RA, Naas T, Dortet L. 2022. Emergence of VIM-producing *Enterobacter cloacae* complex in France between 2015 and 2018. J Antimicrob Chemother 77:944–951. doi:10.1093/jac/dkab471. 35045171

[44] Harada S, Aoki K, Ohkushi D, Okamoto K, Takehana K, Akatsuchi T, Ida K, Shoji D, Ishii Y, Doi Y, Moriya K, Hayama B. 2021. Institutional outbreak involving multiple clades of IMP-producing *Enterobacter cloacae* complex sequence type 78 at a cancer center in Tokyo, Japan. BMC Infect Dis 21:289. doi:10.1186/s12879-021-05952-9. 33752612 PMC7983292

[45] Villa L, García-Fernández A, Fortini D, Carattoli A. 2010. Replicon sequence typing of IncF plasmids carrying virulence and resistance determinants. J Antimicrob Chemother 65:2518–2529. doi:10.1093/jac/dkq347. 20935300

[46] Rozwandowicz M, Brouwer MSM, Fischer J, Wagenaar JA, Gonzalez-Zorn B, Guerra B, Mevius DJ, Hordijk J. 2018. Plasmids carrying antimicrobial resistance genes in Enterobacteriaceae. J Antimicrob Chemother 73:1121–1137. doi:10.1093/jac/dkx488. 29370371

[47] Frost LS, Koraimann G. 2010. Regulation of bacterial conjugation: balancing opportunity with adversity. Future Microbiol 5:1057–1071. doi:10.2217/fmb.10.70. 20632805

[48] Hu F-p, Xu X-g, Zhu D-m, Wang M-g. 2008. Coexistence of *qnrB4* and *qnrS1* in a clinical strain of *Klebsiella pneumoniae*. Acta Pharmacol Sin 29:320–324. doi:10.1111/j.1745-7254.2008.00757.x. 18298896

[49] Partridge SR, Kwong SM, Firth N, Jensen SO. 2018. Mobile genetic elements associated with antimicrobial resistance. Clin Microbiol Rev 31. doi:10.1128/CMR.00088-17. PMC6148190 30068738

[50] Kamruzzaman M, Patterson JD, Shoma S, Ginn AN, Partridge SR, Iredell JR. 2015. Relative strengths of promoters provided by common mobile genetic elements associated with resistance gene expression in gram-negative bacteria. Antimicrob Agents Chemother 59:5088–5091. doi:10.1128/AAC.00420-15. 26055385 PMC4505201

[51] Liu B-T, Yang Q-E, Li L, Sun J, Liao X-P, Fang L-X, Yang S-S, Deng H, Liu Y-H. 2013. Dissemination and characterization of plasmids carrying *oqxAB*-*bla*_CTX-M_ genes in *Escherichia coli* isolates from food-producing animals. PLoS One 8:e73947. doi:10.1371/journal.pone.0073947. 24040123 PMC3767592

[52] Liu X, Liu H, Li Y, Hao C. 2016. High prevalence of *β*-lactamase and plasmid-mediated quinolone resistance genes in extended-spectrum cephalosporin-resistant *Escherichia coli* from Dogs in Shaanxi, China. Front Microbiol 7:1843. doi:10.3389/fmicb.2016.01843. 27899921 PMC5111280

[53] Pu X-Y, Pan J-C, Gu Y-M, Zheng W, Li J, Yu H. 2016. Complete sequences and characterization of two novel plasmids carrying *aac(6')-Ib-cr* and *qnrS* Gene in *Shigella flexneri*. Microb Drug Resist 22:115–122. doi:10.1089/mdr.2015.0082. 26469217

[54] Navas J, Fernández-Martínez M, Salas C, Cano ME, Martínez-Martínez L. 2016. Susceptibility to aminoglycosides and distribution of *aph* and *aac(3)-XI* genes among *Corynebacterium striatum* clinical isolates. PLoS One 11:e0167856. doi:10.1371/journal.pone.0167856. 27936101 PMC5148030

[55] He T, Wei R, Zhang L, Sun L, Pang M, Wang R, Wang Y. 2017. Characterization of NDM-5-positive extensively resistant *Escherichia coli* isolates from dairy cows. Vet Microbiol 207:153–158. doi:10.1016/j.vetmic.2017.06.010. 28757017

[56] Wein T, Hülter NF, Mizrahi I, Dagan T. 2019. Emergence of plasmid stability under non-selective conditions maintains antibiotic resistance. Nat Commun 10:2595. doi:10.1038/s41467-019-10600-7. 31197163 PMC6565834

[57] Sankar TS, Wastuwidyaningtyas BD, Dong Y, Lewis SA, Wang JD. 2016. The nature of mutations induced by replication–transcription collisions. Nature 535:178–181. doi:10.1038/nature18316. 27362223 PMC4945378

[58] Yu R, Xu Y, Schwarz S, Shang Y, Yuan X, Zhang Y, Li D, Du X-D. 2022. *erm*(T)-mediated macrolide-lincosamide resistance in *Streptococcus suis*. Microbiol Spectr 10:e0165721. doi:10.1128/spectrum.01657-21. 35019703 PMC8754144

[59] De Gelder L, Ponciano JM, Joyce P, Top EM. 2007. Stability of a promiscuous plasmid in different hosts: no guarantee for a long-term relationship. Microbiology (Reading) 153:452–463. doi:10.1099/mic.0.2006/001784-0. 17259616

[60] San Millan A, MacLean RC. 2017. Fitness costs of plasmids: a limit to plasmid transmission. Microbiol Spectr 5. doi:10.1128/microbiolspec.MTBP-0016-2017. PMC11687550 28944751

[61] Kizny Gordon A, Phan HTT, Lipworth SI, Cheong E, Gottlieb T, George S, Peto TEA, Mathers AJ, Walker AS, Crook DW, Stoesser N. 2020. Genomic dynamics of species and mobile genetic elements in a prolonged *bla*_IMP-4_-associated carbapenemase outbreak in an Australian hospital. J Antimicrob Chemother 75:873–882. doi:10.1093/jac/dkz526. 31960024 PMC7069471

[62] Gravey F, Cattoir V, Ethuin F, Fabre L, Beyrouthy R, Bonnet R, Le Hello S, Guérin F. 2020. *ramR* deletion in an *Enterobacter hormaechei* isolate as a consequence of therapeutic failure of key antibiotics in a long-term hospitalized patient. Antimicrob Agents Chemother 64. doi:10.1128/AAC.00962-20. PMC7508606 32778545

[63] Wenger PN, Tokars JI, Brennan P, Samel C, Bland L, Miller M, Carson L, Arduino M, Edelstein P, Aguero S, Riddle C, O'Hara C, Jarvis W. 1997. An outbreak of *Enterobacter hormaechei* infection and colonization in an intensive care nursery. Clin Infect Dis 24:1243–1244. doi:10.1086/513650. 9195091

[64] Xu J, Zheng C, Cao M, Zeng T, Zhao X, Shi G, Chen H, Bei W. 2017. The manganese efflux system MntE contributes to the virulence of *Streptococcus suis* serotype 2. Microb Pathog 110:23–30. doi:10.1016/j.micpath.2017.06.022. 28629722

[65] Alquethamy SF, Adams FG, Naidu V, Khorvash M, Pederick VG, Zang M, Paton JC, Paulsen IT, Hassan KA, Cain AK, McDevitt CA, Eijkelkamp BA. 2020. The role of zinc efflux during *Acinetobacter baumannii* infection. ACS Infect Dis 6:150–158. doi:10.1021/acsinfecdis.9b00351. 31658418

[66] Rosch JW, Sublett J, Gao G, Wang Y-D, Tuomanen EI. 2008. Calcium efflux is essential for bacterial survival in the eukaryotic host. Mol Microbiol 70:435–444. doi:10.1111/j.1365-2958.2008.06425.x. 18761687 PMC2577294

[67] Radnedge L, Davis MA, Youngren B, Austin SJ. 1997. Plasmid maintenance functions of the large virulence plasmid of *Shigella flexneri*. J Bacteriol 179:3670–3675. doi:10.1128/jb.179.11.3670-3675.1997. 9171415 PMC179163

[68] Duprilot M, Decre D, Genel N, Drieux L, Sougakoff W, Arlet G. 2017. Diversity and functionality of plasmid-borne VagCD toxin-antitoxin systems of *Klebsiella pneumoniae*. J Antimicrob Chemother 72:1320–1326. doi:10.1093/jac/dkw569. 28119479

[69] Bergsten G, Wullt B, Svanborg C. 2005. *Escherichia coli*, fimbriae, bacterial persistence and host response induction in the human urinary tract. Int J Med Microbiol 295:487–502. doi:10.1016/j.ijmm.2005.07.008. 16238023

[70] Chukwudi CU, Good L. 2020. Doxycycline induces Hok toxin killing in host *E. coli*. PLoS One 15:e0235633. doi:10.1371/journal.pone.0235633. 32628709 PMC7337300

